# Associations between emotional awareness deficits and somatic symptoms in a community and clinical populations: a cross-sectional study

**DOI:** 10.1186/s40359-025-03087-z

**Published:** 2025-07-18

**Authors:** Sunyoung Kang, Chun Il Park, Se Joo Kim, Jee In Kang

**Affiliations:** 1 Institute of Behavioral Science in Medicine, Yonsei University College of Medicine, Seoul, 03722 South Korea; 2 Department of Psychiatry, Yonsei University College of Medicine, Yonsei-ro 50-1, Seodaemun-gu, Seoul, 03722 South Korea

**Keywords:** Emotion, Alexithymia, Empathy, Somatic symptom, Somatic symptom disorder

## Abstract

**Background:**

Deficits in emotional awareness may contribute to the development and maintenance of somatic symptoms. This study explored emotional awareness deficits and their association with somatic symptoms among individuals with a high somatic symptom burden from an online community sample, as well as among patients with somatic symptom disorders.

**Methods:**

Emotional awareness deficits were analyzed by comparing 77 individuals with a high somatic symptom burden and 129 individuals with a low somatic symptom burden from a community population (Study 1). The severity of somatic symptom burden was measured using the Somatic Symptom Scale-8, with scores of eight or higher classified as high. Deficits in emotional awareness in clinical somatic symptoms were examined by comparing 34 patients with somatic symptom disorders to 34 matched healthy controls (Study 2). Emotional awareness was assessed by evaluating alexithymia using the 20-Item Toronto Alexithymia Scale (TAS-20) and empathy using the Interpersonal Reactivity Index (IRI). Multivariate analysis of covariance (MANCOVA) was conducted to examine group differences in emotional awareness while controlling for potential covariates.

**Results:**

After adjusting for covariates, the MANCOVA results in Study 1 revealed significantly higher scores on the Difficulty Identifying Feelings subscale of the TAS-20 and the Personal Distress subscale of the IRI among participants with a high somatic symptom burden. These findings were replicated in Study 2, where patients with somatic symptom disorders exhibited deficits comparable to those of healthy controls.

**Conclusions:**

This study suggests that difficulties in emotional awareness are closely associated with somatic symptoms in both clinical and community populations. Interventions aimed at improving emotional awareness may alleviate the manifestations of somatic symptoms and prevent related functional impairments.

**Clinical trial number:**

Not applicable.

**Supplementary Information:**

The online version contains supplementary material available at 10.1186/s40359-025-03087-z.

## Background

Somatic symptom disorder is characterized by persistent somatic symptoms accompanied by significant psychological distress at the affective, cognitive, and behavioral levels, regardless of whether a medical explanation has been identified [1]. This highlights the central role of psychological mechanisms—particularly emotional dysregulation—in the development and maintenance of the disorder. Prior research has highlighted impairments across various stages of emotion regulation [2–4]. At the identification stage, individuals with somatic symptom disorder often struggle with emotional awareness and clarity [2, 5]. During the selection stage, cognitive factors such as attentional bias, maladaptive somatic beliefs, and misinterpretation of bodily sensations may lead to reliance on defense mechanisms such as repression, suppression, and rumination, rather than on more adaptive strategies such as reappraisal or acceptance [6–8]. Finally, the implementation of these strategies may increase fixation on physical symptoms and exacerbate functional impairments [7, 9]. These emotion regulation deficits are closely linked to central sensitization—a key neural feature of somatic symptom disorder—and are associated with alterations in brain regions including the prefrontal cortex, insula, amygdala, somatosensory cortex, and cingulate cortex [10, 11]. These neural and psychological processes likely interact bidirectionally, reinforcing the persistence and severity of symptoms.

Emotional awareness, which constitutes the initial stage of emotion regulation, has been recognized as a key factor in understanding somatic symptom disorder [12–14]. Individuals with somatic symptoms often remain at early levels of emotional awareness, as described in Lane’s model, where emotions are experienced primarily as bodily sensations or action-oriented urges [15–17]. These difficulties are believed to stem from early insecure attachment, childhood trauma, or past experiences of illness or observing illness behavior [18, 19]. Furthermore, depression has been identified as a mediating factor in the relationship between emotional awareness and somatic distress [20–23]. Given its close association with both constructs, depression is considered a critical factor that may exacerbate deficits in emotional awareness while simultaneously intensifying the perception and reporting of somatic symptoms. Consequently, affected individuals may struggle to advance to more complex levels of emotional functioning, which involve attending to emotional states, achieving clarity, and distinguishing between their own and others’ emotions. Within this framework, the concepts of alexithymia—difficulties in identifying and describing one’s own emotions—and empathy—the ability to understand others’ emotional experiences—have received particular attention [24–28]. Improving these capacities may facilitate the development of mentalization, a more integrated understanding of the mental states of self and others, and help reduce maladaptive responses such as excessive focus on somatic symptoms [17].

Alexithymia, defined as difficulty in identifying and describing one’s own emotions, has been linked to the development, persistence, and poor prognosis of somatic symptoms [29–34]. Recent studies have shown that alexithymia is associated with greater pain intensity and physical symptom interference [32]. It also influences somatic symptoms both directly and indirectly through depressive symptoms [33]. Notably, difficulty in identifying feelings has been identified as a key predictor of somatic symptom severity [34]. This impaired emotional awareness may lead to the somatization of psychological distress, in which unresolved emotional states manifest as physical symptoms. However, several reviews have highlighted methodological limitations in previous research [29–31]. First, most studies did not control for known covariates of alexithymia, such as age, sex, socioeconomic status (SES), education level, depression, and anxiety [29]. Second, many studies were limited to student samples, restricting the range and generalizability of reported somatic symptoms [24–26]. Third, few studies assessed individuals attending psychiatric or medical facilities due to pre-existing conditions, increasing the likelihood of overestimating the association between somatic symptoms and alexithymia in these populations.

Empathy is defined as the ability to understand and share another person’s emotional state [35]. It can be divided into two dimensions: (1) affective empathy (sharing emotions with others) and (2) cognitive empathy (understanding the emotions of others) [36]. However, the relationship between affective empathy and somatic symptoms remains poorly understood. One fMRI study found that, when observing negative facial expressions, patients with somatic symptoms exhibited abnormal activation in limbic and somatosensory brain regions and reported higher anxiety and depression scores compared to healthy controls [37]. This suggests increased susceptibility to emotional contagion in somatic symptom disorders, thereby amplifying emotional distress. Based on previous findings, we hypothesize that individuals prone to somatization would show affective empathy deficits specific to negative emotions. Furthermore, cognitive empathy plays a modulating role in vicarious pain arising from affective empathy [38, 39]. Patients with somatic symptom disorders also perform worse on Theory of Mind tasks (e.g., social animation assessments) than controls [38, 39]. Thus, heightened affective empathy for negative stimuli and impaired cognitive empathy may contribute to somatic distress. However, no studies to date have empirically tested this hypothesis.

This study aimed to investigate difficulties in emotional awareness and their association with somatic symptoms across two distinct populations: individuals with a high somatic symptom burden from an online community sample and patients diagnosed with somatic symptom disorders. Emotional awareness was assessed in terms of two dimensions—alexithymia and empathy. We hypothesized that both individuals with a high somatic symptom burden in the community and patients with somatic symptom disorders would exhibit greater deficits in alexithymia and empathy compared to their respective control groups. To address these hypotheses, Study 1 compared emotional awareness characteristics between individuals with high and low somatic symptom burdens in a community-based sample, while Study 2 examined differences between patients with somatic symptom disorders and healthy controls to determine whether findings from the community sample could be generalized to clinical populations. Additionally, we examined whether these associations remained statistically significant after adjusting for potential covariates such as depression and early life trauma.

## Methods

We designed a two-step study to examine individuals with a high somatic symptom burden and those with clinically diagnosed somatic symptom disorders, focusing on their associations with emotional awareness deficits. In Study 1, differences in emotional awareness subscales were compared between high and low somatic symptom burden groups, categorized based on the Somatic Symptom Scale-8 (SSS-8). Subsequently, in Study 2, a case-control design was used to assess whether the findings from Study 1 could be generalized to individuals diagnosed with somatic symptom disorders compared to age- and sex-matched healthy controls.

### Participants

In Study 1, 207 participants were recruited through a notice posted on the SurveyMonkey website between July 2021 and November 2021. One participant did not complete all study measures and was therefore excluded, leaving 206 participants with complete data. Participants completed an online survey that included self-report questionnaires such as the Center for Epidemiologic Studies Depression Scale (CES-D), 20-Item Toronto Alexithymia Scale (TAS-20), Interpersonal Reactivity Index (IRI), and Childhood Trauma Events Scale (CTES), as well as demographic information (age, sex, education level, and SES). Subsequently, each participant underwent a one-on-one clinical interview with a trained psychologist. The absence of current psychiatric disorders at the time of enrollment was confirmed through the MINI International Neuropsychiatric Interview [40]. Past psychiatric history and physical illness were also assessed. Participants with a lifetime history of schizophrenia spectrum disorder or bipolar disorder, as defined by the DSM-V, as well as those with a history of brain injury, convulsive disorders, or any neurological symptoms, were excluded. Additionally, participants were excluded if they had any current, active, or uncontrolled physical illness requiring ongoing medical intervention or causing significant symptoms at enrollment. Participants in the total sample were aged between 19 and 46 years (mean = 29.1; SD = 6.73) and included 122 women (59.2%).

In Study 2, participants with somatic symptom disorders were recruited between March 2015 and November 2023 at the Specialty Somatic Symptom Disorders Clinic of Severance Hospital, a tertiary referral center in Korea. Diagnoses were made by a trained psychiatrist (J.I. Kang) according to DSM-V criteria [1]. All patients also underwent the MINI International Neuropsychiatric Interview, administered by a trained psychologist, to assess comorbid psychiatric disorders at enrollment [40]. Inclusion in the somatic symptom disorder group required a primary diagnosis of somatic symptom disorder. Healthy controls with no current psychiatric or medical disorders were recruited from an online community through survey advertisements. They first completed online self-report measures (the CES-D, TAS-20, IRI, and CTES) and demographic questionnaires, followed by individual clinical interviews with a trained psychologist. The MINI was administered to confirm the absence of current psychiatric disorders and any lifetime history of somatic symptom disorder [40]. Information on psychiatric and physical health histories was also collected. Exclusion criteria for both groups included a lifetime history of schizophrenia spectrum disorder, bipolar disorder, brain injury, epilepsy, neurological disorders, or any current active or uncontrolled physical illness requiring treatment or causing significant symptoms at enrollment. Participants who did not complete the baseline questionnaires were excluded. Ultimately, 34 patients with somatic symptom disorder and 34 healthy controls, matched for age and sex, were included in Study 2.

All participants included in the study provided written informed consent prior to participation. This study was approved by the Institutional Review Board of Yonsei University Severance Hospital (IRB: 4-2019-0929) and conducted according to the principles of the Declaration of Helsinki.

### Measures of demographic variables and clinical symptoms

Sociodemographic characteristics such as age, sex, education level, SES, and history of physical illness were collected. Education level was categorized as ≤ 12 years (high school or less) or > 12 years (more than high school) [41]. SES was classified as either high or low.

Center for Epidemiologic Studies Depression Scale (CES-D).

Depression was assessed using the CES-D [42], a 20-item self-report scale that assesses depressive symptoms over the past seven days on a 4-point Likert scale (0 to 3). Total scores range from 0 to 60, with higher scores indicating greater severity. The Korean version has demonstrated high reliability and validity in both clinical and nonclinical populations [43]. A score of 24 is generally accepted as indicative of clinically relevant symptoms [44]. In this study, participants who scored 24 or higher on the CES-D were classified as having severe depressive symptoms, while those scoring below 24 were classified as having non-severe depression. The Cronbach’s α for the CES-D in the present sample was 0.874.

Childhood Trauma Events Scale (CTES).

Information regarding childhood adversity (under 17 years) was obtained using the CTES [45]. The domains included the death of a family member or close friend, parental divorce or separation, traumatic sexual experiences, being a victim of violence, being extremely ill or injured, and other major upheavals. Participants were asked whether each type of traumatic event occurred, the age at which it happened, the perceived intensity of the trauma (on a 7-point scale, where 1 = not at all traumatic, 7 = extremely traumatic), and whether the trauma was confided to others. In this study, childhood adversity was identified by responding “yes” to questions about experiencing each trauma type. Participants who responded “yes” to at least one of the six trauma items were classified as having experienced trauma exposure. The Cronbach’s α for the CTES in the present sample was 0.470, reflecting the heterogeneous nature of the items assessing six distinct types of traumatic events.

### Measures of emotional awareness

#### 20-Item Toronto alexithymia scale (TAS-20)

The self-reported 20-item TAS-20 [46, 47] was designed to measure alexithymia, which is the difficulty of being aware of one’s own emotions. Each statement is scored on a 5-point Likert scale ranging from 1 (strongly disagree) to 5 (strongly agree). The total score ranges from 20 to 100, with higher scores indicating greater levels of alexithymia. The TAS-20 has three subscales: Difficulty Describing Feelings (DDF) to others, Difficulty Identifying Feelings (DIF), and Externally Oriented Thinking (EOT). The TAS-20 is known for good test-retest reliability, internal consistency, construct validity, and factor structure, and it is theoretically congruent with alexithymia, similar to the Korean version [48, 49]. The Cronbach’s α of the TAS-20 in the present sample was 0.827.

#### Interpersonal reactivity index (IRI)

Empathy was assessed using the IRI, which evaluates both cognitive and affective empathy [50]. The IRI comprises subscales with seven items each: Perspective Taking (PT), Fantasy (FS), Empathic Concern (EC), and Personal Distress (PD). Each item is rated on a 5-point Likert scale ranging from 1 (does not describe me well) to 5 (describes me very well). The PT subscale measures the tendency to spontaneously adopt others’ psychological perspectives. The FS subscale measures the tendency to transpose oneself into the experiences of fictitious characters. The EC subscale assesses other-oriented emotional responses. The PD subscale measures self-oriented feelings of personal anxiety and unease in tense interpersonal settings. The validity and reliability of the Korean version of the IRI have been previously confirmed [51]. The Cronbach’s α of the IRI in the present sample was 0.808.

### Measures of somatic symptom burden

Somatic symptom burden was measured using the SSS-8 [52]. The SSS-8 consists of eight items reflecting common somatic symptoms in primary care, such as gastrointestinal, musculoskeletal, pain, cardiopulmonary, and fatigue symptoms. Respondents rated how much each symptom had bothered them during the previous seven days and scored each item on a 5-point scale (0 = not at all, 4 = very strongly). Because scores lower than eight indicate lower levels of somatic symptom burden, we used a cutoff score of 8 on the SSS-8 to determine the presence of a high somatic symptom burden [52]. The Korean version of the SSS-8 has demonstrated high validity and reliability [53]. The Cronbach’s α of the SSS-8 in the present sample was 0.842.

### Statistical analyses

All statistical analyses were performed using the Statistical Package for the Social Sciences (SPSS Inc., Chicago, IL, USA) version 26.0. The reliability of all the scales was evaluated using Cronbach’s α. In Study 1, demographic and clinical variables were compared between high and low somatic symptom burden groups using independent t-tests for continuous variables and χ² tests for categorical variables. Multivariate analysis of covariance (MANCOVA) was conducted to compare TAS-20 and IRI subscale scores between groups, controlling for sex, education level, depression severity (classified using a CES-D cutoff of 24), and trauma exposure.

In Study 2, a case-control design using propensity score matching (1:1 ratio for age and sex) was used to compare patients diagnosed with somatic symptom disorders with healthy controls. Demographic and clinical variables were compared between groups using independent t-tests for continuous variables and χ² tests for categorical variables. MANCOVA, adjusted for education level, depression severity, and trauma exposure, was conducted to compare the TAS-20 and IRI subscales between participants with somatic symptom disorders and matched healthy controls.

For both studies, multicollinearity between covariates and the TAS-20 and IRI subscales was assessed using the Variance Inflation Factor (VIF), with VIF < 2.5 indicating acceptable collinearity. The significance level was set at *p* < 0.05. All tests were two-tailed. For the MANCOVA analyses in Studies 1 and 2, Bonferroni-adjusted *p*-values were reported, with *p* < 0.05 also considered statistically significant.

## Results

### Characteristics of the sample in studies 1 and 2

The sociodemographic and clinical characteristics of Study 1 are presented in Table 1. Based on SSS-8 scores, 77 participants were classified into the high somatic symptom burden group (score ≥ 8), and 129 into the low burden group (score < 8). The groups did not differ significantly in terms of age, SES, or history of physical illness. Participants in the high-burden group were more likely to be women (*p* = 0.006) and to have lower education levels (*p* = 0.003). Depression and early life trauma showed significant between-group differences. Specifically, CES-D scores were higher in the high-burden group (mean = 24.7, SD = 9.46) than in the low-burden group (mean = 12.5, SD = 7.70; t = -10.1, *p* < 0.001). Additionally, CTES item analysis revealed a higher prevalence of violence and other major upheavals in the high-burden group. For the TAS-20, total scores were significantly higher in the high-burden group (mean = 53.1, SD = 10.1) than in the low-burden group (mean = 43.9, SD = 9.80; t = -6.53, *p* < 0.001). Regarding psychiatric history, depressive disorders were the most common diagnoses (low burden: *n* = 4, 3.10%; high burden: *n* = 7, 9.09%), while anxiety disorders (low burden: *n* = 3, 2.33%; high burden: *n* = 3, 3.39%) and obsessive-compulsive disorders (high burden: *n* = 1, 1.30%) were less frequent.

The demographic and clinical characteristics of participants in Study 2 are shown in Table 2. As the somatic symptom disorder group and healthy controls were propensity score-matched for age and sex, no significant differences were observed in terms of age (*p* = 0.822) or sex distribution (*p* = 0.331). While education level and SES did not differ between the groups, the somatic symptom disorder group reported a significantly higher prevalence of physical illness history compared to healthy controls (*p* = 0.001). This group also exhibited significantly higher CES-D scores than the healthy controls (*p* < 0.001). Additionally, this group showed significantly higher frequencies in specific CTES subscales, particularly sexual trauma, extreme illness, and others. For alexithymia (TAS-20), total scores were significantly higher in the somatic symptom disorder group (mean = 53.9, SD = 9.97) than in the healthy controls (mean = 44.7, SD = 10.2; t = -3.76, *p* < 0.001). Regarding psychiatric history, depressive disorder was the most common diagnosis in the somatic symptom disorder group (*n* = 8, 23.5%), followed by anxiety disorders (*n* = 4, 11.8%). Among healthy controls, one participant (2.94%) reported a history of obsessive-compulsive disorder.

**Table 1:** Demographic and clinical characteristics of participants in study 1

	Total (*n* = 206)	SSS-8 < 8 (*n* = 129)	SSS-8 ≥ 8 (*n* = 77)	t/χ2	*p*-value
Age, mean (SD)	29.1 (6.73)	28.7 (6.80)	29.6 (6.60)	-0.918	0.360
Sex, n(%)				7.59	0.006
Men	84 (40.8%)	62 (48.1%)	22 (28.6%)		
Women	122 (59.2%)	67 (51.9%)	55 (71.4%)		
Education level, n(%)				5.65	0.003
≤ 12 years	19 (9.22%)	6 (4.65%)	13 (16.9%)		
> 12 years	187 (90.8%)	123(95.3%)	64 (83.1%)		
SES, n(%)				0.448	0.503
Low	56 (27.2%)	33 (25.6%)	23 (29.9%)		
High	150 (72.8%)	96 (74.4%)	54 (70.1%)		
Physical illness history, n(%)				2.71	0.100
Yes	51 (24.8%)	27 (20.9%)	24 (31.2%)		
No	155 (75.2%)	102(79.1%)	53 (68.8%)		
CES-D, mean (SD)	17.2 (10.4)	12.5(7.70)	24.7(9.46)	-10.1	< 0.001
CTES, n(%)					
Death of a family member or friend	37 (18.0%)	21 (16.3%)	16 (20.8%)	0.663	0.416
Divorce or separation	30 (14.6%)	14 (10.9%)	16 (20.8%)	3.82	0.051
Sexual trauma	18 (8.74%)	6 (4.65%)	12 (15.6%)	7.23	0.070
Violence	17 (8.25%)	6 (4.65%)	11 (14.3)	5.91	0.015
Extreme illness	28 (13.6%)	16 (12.4%)	12 (15.6%)	0.416	0.519
Other	57 (27.7%)	29 (22.5%)	28 (36.4%)	4.64	0.031

SSS-8, Somatic Symptom Scale-8; SES, socioeconomic status; CES-D, Center for Epidemiologic Studies Depression Scale; CTES, Childhood Trauma Events Scale

**Table 2:** Demographic and clinical characteristics of participants in study 2

	Total (*n* = 68)	HC (*n* = 34)	SSD (*n* = 34)	t/χ2	*p*-value
Age, mean (SD)	30.1 (8.52)	29.9 (8.51)	30.3 (8.64)	-0.226	0.822
Sex, n(%)				0.944	0.331
Men	36 (52.9%)	20 (58.8%)	16 (47.1%)		
Women	32 (47.1%)	14 (41.2%)	18 (52.9%)		
Education year, n(%)				0.078	0.779
≤ 12 years	17 (25.0%)	8 (23.5%)	9 (26.5%)		
> 12 years	51 (75.0%)	26 (76.5%)	25 (73.5%)		
SES, n(%)				0.073	0.787
Low	19 (27.9%)	10 (29.4%)	9 (47.4%)		
High	49 (72.1%)	24 (70.6%)	25 (73.5%)		
Physical illness history, n(%)				11.6	0.001
Yes	21 (30.9%)	4 (11.8%)	17 (50.0%)		
No	47 (69.1%)	30 (88.2%)	17 (50.0%)		
CES-D, mean (SD)	22.1 (5.90)	18.9 (3.92)	25.3 (5.87)	-5.27	< 0.001
CTES, n(%)					
Death of a family member or friend	16 (23.5%)	7 (20.6%)	9 (26.5%)	0.327	0.567
Divorce or separation	6 (8.82%)	3 (8.82%)	3 (8.82%)	0.000	1.000
Sexual trauma	4 (5.88%)	0 (0.000%)	4 (11.8%)	4.25	0.039
Violence	6 (8.82%)	1 (2.94%)	5 (14.7%)	2.93	0.087
Extreme illness	14 (20.6%)	2 (5.88%)	12 (35.3%)	8.90	0.003
Other	17 (25.0%)	4 (11.8%)	13 (38.2%)	6.35	0.012

HC, Healthy Control; SSD, Somatic Symptom Disorder; SES, Socioeconomic Status; CES-D, Center for Epidemiologic Studies Depression Scale; CTES, Childhood Trauma Events Scale

### Differences in emotional awareness according to somatic symptom burden in study 1

To assess the independence of the covariates and subscales of the TAS-20 and IRI in Study 1, VIFs were calculated. VIF values for all variables ranged from 1.03 to 2.16, which are below the commonly accepted threshold of 2.5, indicating negligible multicollinearity. As shown in Table 3, the MANCOVA results indicated significant associations between emotional awareness measures and somatic symptom burden severity (TAS-20: F = 10.8, df = 3,198, *p* < 0.001; IRI: F = 2.86, df = 4,197, *p* = 0.025). For alexithymia, participants with a high somatic symptom burden exhibited significantly higher DIF scores compared to the low-burden group (Bonferroni-adjusted *p* < 0.001). No group differences were observed for DDF or EOT. Regarding empathy, the high-burden group showed elevated PD scores (Bonferroni-adjusted *p* = 0.008). No differences were found in PT, FS, or EC between the groups.

**Table 3:** MANCOVA of TAS-20 and IRI between individuals with high and low somatic symptom burden in study 1

TAS-MANCOVA	SSS-8 < 8 (*n* = 129)	SSS-8 ≥ 8 (*n* = 77)	Wilks’s lamda = 0.859, F = 10.8, df = 3,198, Partial η² = 0.141, *p* < 0.001		
	Mean (SD)	Mean (SD)	F	Partial η²	Bonferroni-adjsuted *p*-value
DDF	12.1 (4.32)	14.2 (3.98)	2.49	0.012	0.348
DIF	12.6 (5.01)	18.9 (5.66)	27.9	0.122	< 0.001
EOT	19.1 (3.43)	20.0 (3.70)	0.546	0.003	1.00

IRI-MANCOVA	SSS < 8 (*n* = 129)	SSS ≥ 8 (*n* = 77)	Wilks’s lamda = 0.945, F = 2.86, df = 4,197, Partial η² = 0.055, *p* = 0.025		
	Mean (SD)	Mean (SD)	F	Partial η²	Bonferroni-adjsuted *p*-value
PT	16.6 (3.97)	16.1 (4.07)	0.099	< 0.001	1.00
FS	15.7 (5.27)	17.6 (5.48)	2.64	0.013	0.424
EC	16.2 (4.28)	16.7 (4.76)	3.12	0.015	0.316
PD	12.8 (4.35)	16.3 (4.59)	9.72	0.046	0.008

SSS-8, Somatic Symptom Scale-8; TAS-20, 20-Item Toronto Alexithymia Scale; DDF, Difficulty Describing Feelings; DIF, Difficulty Identifying Feelings; EOT, Externally Oriented Thinking; IRI, Interpersonal Reactivity Index; PT, Perspective Taking; FS, Fantasy; EC, Empathic Concern; PD, Personal Distress; MANCOVA, Multivariate Analysis of Covariance (covariates: sex, education level, depression severity, and trauma exposure)

### Group differences in somatic symptom burden due to alexithymia and empathy in study 2

To assess the independence of the covariates and subscales of the TAS-20 and IRI in Study 2, VIFs were calculated. VIF values for all variables ranged from 1.03 to 1.76, which are below the commonly accepted threshold of 2.5, indicating negligible multicollinearity. As shown in Table 4, the MANCOVA results revealed significant differences in the TAS-20 subscales between the two groups (F = 5.31, df = 3, 61, *p* = 0.003). Specifically, patients with somatic symptom disorders exhibited significantly higher DIF scores compared to healthy controls (Bonferroni-adjusted *p* = 0.009), while no significant differences were observed for DDF or EOT. For IRI, the comparison between the somatic symptom disorder group and healthy controls showed significant differences (F = 3.35, df = 4, 60, *p* = 0.015). Subscale analysis revealed significantly higher PD scores in the somatic symptom disorder group (Bonferroni-adjusted *p* = 0.016), with no group differences in PT, FS, or EC.

**Table 4:** MANCOVA of TAS-20 and IRI between patients with somatic symptom disorder and healthy controls in study 2

TAS-MANCOVA	HC	SSD	Wilks’s lamda = 0.793, F = 5.31, df = 3, 61, Partial η² = 0.207, *p* = 0.003		
	Mean (SD)	Mean (SD)	F	Partial η²	Bonferroni-adjusted *p*-value
DDF	11.2 (4.12)	12.9 (4.80)	0.774	0.012	1.00
DIF	12.2 (4.99)	19.2 (6.01)	9.78	0.134	0.009
EOT	21.4 (3.28)	21.9 (4.10)	0.782	0.012	1.00

IRI-MANCOVA	HC	SSD	Wilks’s lamda = 0.818, F = 3.35, df = 4, 60, Partial η² = 0.182, *p* = 0.015		
	Mean (SD)	Mean (SD)	F	Partial η²	Bonferroni-adjusted *p*-value
PT	18.4 (4.18)	16.4 (4.31)	1.03	0.016	1.00
FS	15.4 (6.47)	15.9 (4.96)	0.123	0.002	1.00
EC	16.5 (5.12)	17.2 (5.26)	3.99	0.060	0.200
PD	11.3 (5.55)	15.7 (5.21)	8.79	0.125	0.016

HC, Healthy Control; SSD, Somatic Symptom Disorder; TAS-20, 20-Item Toronto Alexithymia Scale; DDF, Difficulty Describing Feelings; DIF, Difficulty Identifying Feelings; EOT, Externally Oriented Thinking; IRI, Interpersonal Reactivity Index; PT, Perspective Taking; FS, Fantasy; EC, Empathic Concern; PD, Personal Distress; MANCOVA, Multivariate Analysis of Covariance (covariates: educational level, depression severity, and trauma exposure)

## Discussion

This study investigated characteristics of emotional awareness—alexithymia and empathic ability—in relation to high somatic burden and clinical somatic symptoms. Groups with a high somatic symptom burden showed elevated scores on the DIF subscale of the TAS-20, reflecting core deficits in identifying one’s own emotions. Regarding empathy, individuals with high somatic distress exhibited heightened PD in response to others’ negative emotions; however, no significant differences were observed in PT, FS, or EC. These findings were consistent with those of Study 2, in which individuals with clinically diagnosed somatic symptom disorders showed similar emotional awareness deficits compared to age- and sex-matched healthy controls. The findings of this study suggest that difficulties in emotional awareness are transdiagnostic markers linked to somatic symptom burden across both clinical and community populations.

To the best of our knowledge, this study is the first to analyze the association between emotional awareness (encompassing alexithymia and empathy) and somatic symptoms across both community samples and clinical populations. A review article including 16 studies on the association between alexithymia and somatic symptoms highlighted that most prior studies failed to control for confounding factors such as demographic and clinical variables [29]. In the present study, covariates including sex, education level, depression severity, and trauma exposure were controlled for in the MANCOVA. After adjusting for these confounders, our findings demonstrated that individuals with emotional awareness deficits are more vulnerable to somatic symptoms compared to those without such deficits.

Individuals with high somatic symptom burden and somatic symptom disorder were associated with greater deficits in alexithymia compared to those without, particularly in the ability to identify their emotions. Consistent with previous studies, these findings support the association between alexithymia, as measured by the TAS-20, and somatic symptoms [32, 54–56]. Numerous studies have suggested that individuals with alexithymia are more likely to misinterpret emotionally driven physiological sensations, leading to hypochondriacal concerns and somatization [56–59]. This relationship is further supported by biological evidence linking alexithymia to increased activation of the sympathetic nervous system and the hypothalamic-pituitary-adrenal axis, as well as elevated levels of inflammatory cytokines such as IL-6 [60, 61]. Neurologically, reduced functional connectivity between the prefrontal cortex and amygdala has been reported, potentially affecting both emotional regulation and bodily perception. In particular, fMRI studies have shown decreased anterior insular activation—a region directly implicated in interoception—in individuals with alexithymia, further supporting the link between alexithymia and somatic symptoms [54, 62]. Specifically, in both Studies 1 and 2, the DIF scores remained significantly higher in the high somatic symptom burden and somatic symptom disorder groups, whereas no significant differences were observed in the DDF or EOT. Studies have found that among the TAS-20 subscales, DIF exhibits the strongest association with somatic symptoms, while findings regarding DDF and EOT have been controversial [29, 34, 63, 64]. Studies assessing the association between somatosensory amplification—the tendency to perceive somatic sensations as intense, noxious, or alarming—and alexithymia have found that individuals with high DIF scores tend to misinterpret somatic symptoms as threatening [65, 66]. Therefore, it can be hypothesized that the inability to identify emotions may lead to hypochondrial concerns regarding negative affect-related bodily sensations, thereby exacerbating the somatic symptom burden.

Regarding empathy, when comparing high somatic symptom burden with low somatic symptom burden, only the PD group scored significantly higher, while no group differences were observed among the other IRI subscales. This pattern was consistent even among patients with somatic symptom disorders. Although direct comparisons are limited due to scarce research on IRI-assessed empathy and somatic symptoms, studies suggest that excessive affective empathy is a risk factor for somatic symptoms, whereas cognitive empathy modulates symptom severity [39, 67, 68]. Individuals with somatic symptoms have been found to exhibit abnormal brain activity, impaired emotion recognition, and elevated empathic distress—characterized by anxiety and discomfort in response to others’ negative emotions [37]—implying that heightened affective empathy may drive somatic symptom expression. For example, among college students, higher affective empathy was linked to greater internalizing symptoms, mediated by emotion regulation difficulties [68]. These findings underscore the emotional toll of excessive affective empathy when regulatory mechanisms are deficient. Among the IRI subscales, PT and FS assess cognitive empathy, whereas EC and PD measure affective empathy [69]. In this study, significant differences were observed between the two groups only in PD, indicating that excessive affective empathy particularly contributes to the somatic symptom burden. In affective empathy, EC refers to the outward process of empathizing with others’ emotions, while PD involves the inward experience of others’ negative emotions. Thus, individuals with high somatic symptom burdens tend to exhibit elevated levels of PD, suggesting that they experience heightened vicarious internal emotional arousal in response to others’ negative emotions [50]. This tendency often leads them to focus inward on themselves, including their bodily sensations, rather than outward toward others [69, 70].

Deficits in emotional awareness have significant treatment implications. These deficits lead to maladaptive behaviors in response to somatic symptoms. Early interventions targeting these deficits could alleviate pain and improve social functioning. Cognitive Behavioral Therapy (CBT), which involves tracking pain intensity and emotional states and correcting maladaptive automatic thoughts, has led to improvements in pain, depression, anxiety, and pain catastrophizing among patients with somatoform disorder [70]. Similarly, mindfulness-based CBT for patients with severe chronic pain has been shown to decrease scores on the IRI-PD subscale after treatment [71]. Such CBT interventions enable patients to repeatedly identify their emotions, potentially reducing alexithymic characteristics related to somatic symptom burden, as proposed in this study. Beyond CBT, short-term psychodynamic psychotherapy approaches—including Emotional Awareness and Expression Therapy (EAET) and Intensive Short-Term Dynamic Psychotherapy (ISTDP)—have also shown promise in treating somatic symptom presentations by focusing on emotional processing, trauma resolution, and exploration of unconscious conflicts [72, 73]. In a randomized controlled trial involving patients with somatic symptom disorder, internet-based EAET resulted in significantly greater reductions in somatic symptom severity and psychological distress at post-treatment than a waitlist control group [41]. Notably, improvements in emotional processing were found to mediate treatment outcomes. In another study, adjunctive ISTDP led to moderate reductions in somatic symptom burden and psychological distress in patients with treatment-resistant somatic symptom presentations [74]. Through intensive affective work and the breakdown of defenses, ISTDP facilitates the experience and expression of avoided emotions, which may reduce the intensity and persistence of somatic symptoms. Taken together, these findings suggest that early incorporation of emotion-focused approaches may be particularly beneficial for individuals vulnerable to somatic symptoms, especially when emotional awareness deficits are prominent.

Individuals with a high somatic symptom burden exhibited significantly higher rates of childhood trauma, including violence, sexual trauma, and severe illness. These trauma-related factors may exacerbate deficits in emotional awareness. Research indicates that trauma-exposed youth exhibit impaired emotional conflict regulation, characterized by reduced dorsolateral prefrontal cortex activation and disrupted amygdala-pregenual anterior cingulate cortex inhibitory circuitry [75]. Furthermore, childhood trauma is positively correlated with total TAS-20 scores and its DIF subscale, particularly driven by early life emotional neglect [76, 77]. Childhood Trauma Questionnaire subscale scores were also significantly correlated with PT, PD, and impaired recognition of angry facial emotions [76]. This suggests that early adversity contributes to the development of alexithymic traits and interpersonal tension, thereby perpetuating functional impairment in populations with somatic symptoms.

This study has several limitations. First, clinical variables were assessed cross-sectionally, and no longitudinal follow-up was conducted to observe changes over time. Therefore, a causal relationship between emotional awareness and somatic symptoms could not be established. However, the finding that emotional awareness deficits associated with a high somatic symptom burden in the online community sample (Study 1) were also observed in the clinical patient group (Study 2) indirectly suggests that these deficits may persist at the subclinical level prior to the diagnosis of somatic symptom disorder. As empathy and alexithymia are considered stable personality traits [78, 79], deficits in emotional awareness may contribute to the development and maintenance of somatic symptoms, rather than being solely a consequence of maladaptive responses to these symptoms. Therefore, future prospective studies are required to determine whether emotional awareness deficits predispose individuals to somatic symptom disorders. Second, this study relied on self-reported measures for several demographic and clinical variables, including SES, alexithymia, empathy, and somatic symptom burden. Therefore, these findings should be interpreted with caution, as subjective bias or limited self-awareness may have influenced the responses. Future studies could benefit from a multimethod approach incorporating clinical interviews and objective assessments of emotional states. Third, potential recruitment bias should be considered. In both Study 1 and the healthy control group in Study 2, participants were recruited via online advertisements, which may have skewed the sample toward younger individuals and those with higher educational attainment who are more familiar with digital platforms. Additionally, self-selection bias is possible, as individuals with greater awareness of their physical or emotional symptoms may have been more likely to participate.

## Conclusions

In conclusion, both a high somatic symptom burden and clinical somatic symptoms were associated with difficulties in identifying emotions and with elevated personal distress in interpersonal situations. These findings suggest that deficits in emotional awareness may represent a heightened risk for somatic symptoms. Such deficits may predispose vulnerable individuals to difficulties coping with negative emotions and to excessive preoccupation with bodily sensations. Therefore, early interventions targeting emotional awareness difficulties may help prevent the functional impairments associated with somatic symptoms.

## Electronic supplementary material

Below is the link to the electronic supplementary material.

Supplementary Material 1

## Data Availability

All data generated or analysed during this study are included in this published article and its supplementary information files.

## Abbreviations

CES-D: Center for Epidemiologic Studies Depression Scale
CBT: Cognitive Behavioral Therapy
CTES: Childhood Trauma Events Scale
DDF: Difficulty Describing Feelings
DIF: Difficulty Identifying Feelings
EAET: Emotional Awareness and Expression Therapy
EC: Empathic Concern
EOT: Externally Oriented Thinking
FS: Fantasy
IRI: Interpersonal Reactivity Index
ISTDP: Intensive Short-Term Dynamic Psychotherapy
MANCOVA: Multivariate Analysis of Covariance
PD: Personal Distress
PT: Perspective Taking
SES: Socioeconomic Status
SPSS: Statistical Package for the Social Sciences
SSS-8: Somatic Symptom Scale-8
TAS-20: 20-Item Toronto Alexithymia Scale
VIF: Variance Inflation Factor
